# Increasing temperature elevates the variation and spatial differentiation of pesticide tolerance in a plant pathogen

**DOI:** 10.1111/eva.13197

**Published:** 2021-02-02

**Authors:** Yahuza Lurwanu, Yan‐Ping Wang, E‐Jiao Wu, Dun‐Chun He, Abdul Waheed, Oswald Nkurikiyimfura, Zhen Wang, Li‐Ping Shang, Li‐Na Yang, Jiasui Zhan

**Affiliations:** ^1^ Key Lab for Biopesticide and Chemical Biology Ministry of Education Fujian Agriculture and Forestry University Fuzhou China; ^2^ Department of Crop Protection Faculty of Agriculture Bayero University Kano Nigeria; ^3^ Jiangsu Key Laboratory for Horticultural Crop Genetic Improvement Institute of Pomology Jiangsu Academy of Agricultural Sciences Nanjing China; ^4^ School of Economics and Trade Fujian Jiangxia University Fuzhou China; ^5^ Southern Potato Center of China Enshi Academy of Agricultural Sciences Enshi China; ^6^ Institute of Oceanography Minjiang University Fuzhou China; ^7^ Department of Forest Mycology and Plant Pathology Swedish University of Agricultural Sciences Uppsala Sweden

**Keywords:** adaptive evolution, climatic change, disease management, fitness penalty, fungicide resistance, *Phytophthora infestans*

## Abstract

Climate change and pesticide resistance are two of the most imminent challenges human society is facing today. Knowledge of how the evolution of pesticide resistance may be affected by climate change such as increasing air temperature on the planet is important for agricultural production and ecological sustainability in the future but is lack in scientific literatures reported from empirical research. Here, we used the azoxystrobin‐*Phytophthora infestans* interaction in agricultural systems to investigate the contributions of environmental temperature to the evolution of pesticide resistance and infer the impacts of global warming on pesticide efficacy and future agricultural production and ecological sustainability. We achieved this by comparing azoxystrobin sensitivity of 180 *P*.* infestans* isolates sampled from nine geographic locations in China under five temperature schemes ranging from 13 to 25°C. We found that local air temperature contributed greatly to the difference of azoxystrobin tolerance among geographic populations of the pathogen. Both among‐population and within‐population variations in azoxystrobin tolerance increased as experimental temperatures increased. We also found that isolates with higher azoxystrobin tolerance adapted to a broader thermal niche. These results suggest that global warming may enhance the risk of developing pesticide resistance in plant pathogens and highlight the increased challenges of administering pesticides for effective management of plant diseases to support agricultural production and ecological sustainability under future thermal conditions.

## 1 INTRODUCTION

Continuing evolutionary processes in pathogens can quickly reduce the efficacy of all classes of pesticides after they are commercially adopted for a certain period of time, thereby presenting a great threat to plant disease management in both agricultural and natural ecosystems. Development of pesticide resistance, driven by adaptive pathogen mutations that decrease the binding of the pesticide to target proteins, enhance expression of the target proteins, increase expression of efflux pumps (Avenot et al., 2009; Webber & Piddock, 2003), or change in bio‐property of cell walls (Nikolaidis et al., 2014), is regulated by the intensity and duration of their application (Chen et al., 2013), modes of action (Gisi & Sierotzki, 2008; Lucas et al., 2015), ways of being administered (Zhan et al., 2006), and the evolutionary capacity of the associated pathogens. For example, it is expected that the evolutionary risk of developing resistance against site‐specific pesticides is higher than that against site‐nonspecific pesticides because the development of resistance to the former usually involves only a single amino acid change in the target gene (Chen et al., 2009, 2018; Gisi & Sierotzki, 2008), while in the latter, resistance requires a sequential accumulation of multiple sequence substitutions in many independent genes over the pathogen genome (Mohd‐Assaad et al., 2016; Zhan et al., 2006). In pathogens, growth strategy, reproductive mode, transmission, and the extent of spatiotemporal distribution of genetic variation are among the key biological and population genetic features affecting their potential of developing pesticide resistance (Lucas et al., 2015). In addition, evolution of pesticide resistance is also affected by the ecological interaction of pathogens with other biotic and abiotic factors in the environments (Delnat et al., 2019; Maino et al., 2018), leading to substantial variation in efficacy durability among pesticides (MacFadden et al., 2018).

Temperature is among the most important environmental factors that can have a critical influence on all aspects of biological (Clarke, 2003; Knies et al., 2006), ecological (Chen et al., 2017; Loehle et al., 2016), and biochemical processes of species (Park et al., 2011; Yu et al., 2019). It can affect the development and evolution of pesticide resistance directly by (i) altering chemical properties of pesticide compounds (Schade et al., 2014), (ii) changing the mutation rate and expression of target genes as well as the interaction of the target genes with other genes (Cuco et al., 2018), or (iii) or modifying the enzymatic activities, metabolic rates, and physiological conditions of cells (Mariette et al., 2016; Sharma et al., 2011). Temperature may also have an indirect effect on the evolution of pesticide resistance by regulating the life cycle, density, and population genetic structure of pathogens (Hoffmann & Sgro, 2011; Tooley et al., 2009). Indeed, it has been documented experimentally that air temperature is an important environmental factor that can strongly affect the sensitivity of pathogens to pesticides and fitness of resistant mutants (Rodríguez‐Verdugo et al., 2013; Zhang et al., 2015). In this way, temperature can change the evolutionary landscape of pesticide resistance in plant pathogens (He et al., 2018; Lurwanu et al., 2020; Mohd‐Assaad et al., 2016; Qin et al., 2016).

Average air temperatures on earth have increased by ~1.0°C, and the pattern of change is expected to be escalated in coming decades (IPCC, 2014). In *Escherichia coli*, elevated temperature increased the fitness of mutants with resistance to rifampicin under nutrient deficiency (Rodríguez‐Verdugo et al., 2013), suggesting that increasing air temperature by global warming may facilitate the evolution of pesticide resistance in the bacteria. Increasing temperature also increases pesticide resistance in other infectious human pathogens such as *Klebsiella pneumoniae* and *Staphylococcus aureus* (MacFadden et al., 2018). Such documented results on the impact of increasing temperature on pesticide resistance are primarily derived from short‐term responses of pathogens to a narrow window of temperature fluctuation at the genic or genotypic level. Knowledge of the long‐term effect of temperature on the evolution of pesticide resistance at the organismal level by comparing thermal response patterns of geographically adapted pathogen populations to changing temperature is limited but is important to predict the impact of global warming on the management of agricultural and natural ecosystems and design effective programs for mitigation. In this study, we used the azoxystrobin‐*Phytophthora infestans* interaction in an agricultural system as a model to investigate the contribution of local thermal conditions to the evolution of pesticide resistance in pathogen populations. In particular, we infer the potential impact of ongoing global warming on the development of pesticide resistance and implications of the impact for future infectious disease and ecosystem managements by comparing the response of azoxystrobin sensitivity in *P*.* infestans* originating from different thermal zones to the change of experimental temperatures.

Azoxystrobin is a broad‐spectrum, systemic fungicide commonly used around the world to manage many plant pathogens in agriculture and forestry. It is the leading synthetic pesticide in the strobilurin family derived from a group of natural products (Bartlett et al., 2002). The pesticide suppresses mitochondrial respiration of pathogens by binding its active compound to Qo in the cytochrome *bc_1_* enzyme complex (Complex III), thereby crippling various biological and biochemical processes of living cells by blocking electron transfer, stopping adenosine triphosphate synthesis, and disrupting energy circulation (Du et al., 2019; Thind, 2012). Azoxystrobin was first launched in 1996, but resistant pathotypes emerged shortly after its commercialization (Bartlett et al., 2002). In 1998, the first azoxystrobin resistance was observed in a field population of the wheat powdery mildew pathogen *Blumeria graminis* sampled from northern Germany (Heaney et al., 2000). Since then, field resistance to azoxystrobin has been reported in many plant pathogens globally (FRAC, 2012).

*Phytophthora infestans* (Mont.) de Bary, the causal agent of the 1840s Irish potato famine, is one of the most damaging and economically important plant diseases in the world (Fry, 2008). Under favorable climatic conditions, late blight can destroy an entire potato crop within a week, causing approximately 8 billion US dollars economic losses annually worldwide (Birch et al., 2012; Runno‐Paurson et al., 2013). Pesticide application is one of the most effective approaches to control the disease (Kessel et al., 2018; Rekanović et al., 2012), but resistance to pesticides such as metalaxyl can quickly develop (Chen, Zhou, Xi, et al., 2018; Matson et al., 2015). *P*.* infestans* predominantly reproduces asexually via sporangia that are mostly dispersed by wind and rain (Tian et al., 2015), but sexual cycles have been documented recently in many countries (Danies et al., 2014; Zhu et al., 2015) after the spread of the A2 mating type from Mexico (Flier et al., 2007; Guo et al., 2010) and emergence of self‐fertile pathotypes (Han et al., 2013; Zhu et al., 2016). High genetic variation and enhanced persistence of the pathogen (Hwang et al., 2014; Mayton et al., 2000) associated with sexual reproduction increase its evolutionary potential to adapt to environmental stresses including pesticide application.

The specific objectives of the study are to: (i) understand the variation and spatial distribution of azoxystrobin tolerance in *P*.* infestans*; (ii) determine the main evolutionary mechanism responsible for the variation and distribution of azoxystrobin tolerance in *P*.* infestans*; (iii) evaluate the contribution of temperature on the evolution of azoxystrobin resistance in *P*.* infestans*; and (iv) infer the impact of global warming on the sustainable management of plant disease in agricultural and natural ecosystems.

## 2 MATERIALS AND METHODS

### 2.1 *Phytophthora infestans* collections

Diseased leaves with *P. infestans* symptoms were collected from nine potato fields, along a climatic gradient representing several potato cropping zones in China during the 2010 and 2011 growing seasons (Table S1). Gansu, Guizhou, Hubei, Inner Mongolian, Ningxia, and Yunnan represent the six most intensive potato production areas in China, while Guangxi, Fuzhou, and Xiapu, located in the winter cropping region, are the areas with the greatest possibility of developing a potato industry in coming decades, primarily driven by government promotion and changes in dietary patterns in China. For all collections, infected leaves were sampled at random from potato plants in 1–2 m apart and transported in separate sandwich bags to the laboratory within 24 h for *P*.* infestans* isolation. To isolate the pathogen, infected leaves were first rinsed gently using running tap water and then sterilized distilled water. A small portion of tissue was cut from the advance margin of a leaf lesion and placed abaxial side up on 2.0% water agar for 20–30 h. A single piece of mycelium was removed aseptically from a sporulating lesion using an inoculating needle, transferred to a rye B agar plate supplemented with ampicillin (100 μg/ml) and rifampicin (10 μg/ml), and incubated at 19°C in the dark for 7 days to develop a colony. Purification was performed by three sequential transfers of a single sporangium collected from hyphae to a fresh rye B plate. The isolates were maintained at 4°C on media until use. *P*.* infestans* could lose viability such as pathogenicity after long‐term storage on media (Jinks & Grindle, 1963), and these biological features will be restored when necessary by infecting a susceptible potato cultivar. Details of pathogen collection, isolation and restoration are described in previous publications (Qin et al., 2016; Yang et al., 2016; Zhu et al., 2015).

### 2.2 *Phytophthora infestans* genotyping

Genotypic data of the isolates collected from Ningxia, Gansu, Guizhou, Yunnan, Xiapu, Fuzhou, and Guangxi were taken from previous publications (Wu et al., 2016; Yang et al., 2018; Zhu et al., 2016), while those for the Inner Mongolian and Hubei populations were generated de novo using the same procedures. Briefly, genomic DNA was extracted using a Plant gDNA Miniprep Kit (GD 2611; Biomiga) based on the manufacturer's instructions and amplified with eight pairs of simple sequence repeats (SSR) primers (G11, Pi02, Pi04, Pi4B, Pi16, Pi33, Pi56, and Pi89) developed previously (Knapova & Gisi, 2002; Lees et al., 2006) and labeled with fluorescent dyes (Zhu et al., 2015). PCR amplification was performed in a 25 μl volume in a microtube containing 1.0 μl of *P*.* infestans* genomic DNA (~20 ng), 12.5 μl of 2× PCR Buffer Mix (TransGen Biotech Co., Ltd.), 1.0 μM each of forward and reverse primers in a 2720 thermal cycler (Applied Biosystems) with the following conditions: initiated with a cycle of 2 min at 94°C, followed by 35 cycles of 30 s at 94°C, 25 s at 56–58°C (depending on the primers), and 60 s at 72°C, and finished with an elongation cycle of 5 min at 72°C. The PCR products were loaded into 96‐well plates and sent to Ruiboxingke Biotechnology Company Limited to determine fragment sizes using an ABI 3730XL automated DNA sequencer (Applied Biosystems) in which a DNA size ladder was included in each of the samples (Zhu et al., 2015). Alleles were allocated using GeneMarker software version 3.7 with a binning procedure. Fragments with different sizes generated by a same pair of primers were classified as alleles.

### 2.3 Experimental test for azoxystrobin tolerance

A multilocus genotype was generated for each isolate by joining alleles at each SSR locus in the same order (Zhan & McDonald, 2011; Zhu et al., 2015). A total of 180 *P*.* infestans* isolates, each with a distinct multilocus genotype (20 from each of the nine populations), were selected for the fungicide experiment using the common garden design (Schwaegerle et al., 2000; Zhan et al., 2005; Zhan & McDonald, 2011). Prior to the experiment, isolates maintained at 4°C on media for long‐term storage were revived on rye B agar at 19°C for 8 days. Mycelia plugs (5 mm in diameter) were then taken from the margin of revived colonies and inoculated onto new rye B plates with (treatments) or without (controls) azoxystrobin (Sigma, Aldrich) amendment in 9 cm Petri dishes. Three azoxystrobin concentrations (0.05, 0.10, and 0.30 μg/ml) were used in the experiment and controls (without azoxystrobin) were included in each fungicide treatment (concentration). The azoxystrobin was first dissolved in methanol to make a stock solution and then diluted to the required concentrations. Inoculated plates were exposed to one of the five experimental temperatures (13, 16, 19, 22, and 25°C) in an incubator and were laid out in a completely randomized design using three replications as recommended previously (Yang et al., 2016; Zhan et al., 2006). This thermal range was selected to represent the temperatures the pathogen populations were exposed to during the potato growing phase. Most areas where potato is grown in the world fall within this thermal ranges (Haverkort, 1990). Image analysis software ASSESS (Lamari, 2002) was used to measure the colony sizes starting from day three after inoculation until the eighth day after inoculation.

### 2.4 Data analysis

The growth rate of the *P*.* infestans* isolates in azoxystrobin treatments and controls was estimated using a logistic model (Aguayo et al., 2014) based on the sizes of an individual colony measured at each time over the concentrations in each of five temperatures over the 3–8 day postinoculation periods. Azoxystrobin tolerance, the ability of *P*.* infestans* to survive in the existence of the fungicide, was measured by relative growth rate (RGR) of the pathogen isolates in the presence to the absence of the fungicide (Brunner et al., 2016; Zhan et al., 2006). The tolerance was estimated separately for each fungicide concentration and isolate, and analysis of variance for the tolerance was performed using the general linear model procedure (GLM) embedded in SAS 9.1.3. The thermal reaction norm of azoxystrobin tolerance in each isolate was fitted to a second order polynomial distribution using the RGRs estimated from each of the three fungicide concentrations. The resulted norms were used to compute the maximum (*T*_max_), optimum (*T*_opt_) and minimum (*T*_min_) temperatures, and the maximum tolerance of the isolates for each fungicide concentration as described previously. The thermal reaction norm of azoxystrobin tolerance was also evaluated using the mean RGR of the isolates in a population over all concentrations. Least significant difference was used to compare the azoxystrobin tolerance, *T*_max_, *T*_opt_, *T*_min_, and *T*_breadth_ (*T*_max_ ‐ *T*_min_) among the nine *P*.* infestans* populations (Kokalis‐Burelle et al., 2013) in each fungicide concentration.

Phenotypic variance for azoxystrobin tolerance was estimated and portioned into sources attributed to isolate (I, random effect), population (P, random effect), temperature (T, fixed effect), and fungicide concentration (C, fixed effect) using SAS GLM and VARCOMP programs (SAS 9.1.3) according to the model:

$$Y_{\text{riptc}}=M+I\;\left(P\right)+T+C+P+I\;\left(P\right)*T+I\;\left(P\right)*C+E_{\text{riptc}}$$

where Y_riptc_ refers to the fungicide tolerance of isolate *i* in replicate r, population p at temperature t and concentration c; M is the overall mean; T is the experimental temperature; C is the fungicide concentration; and E_ripc_ is experimental error. The terms P, I (P), I(P)*T, and I(P)*C refer to genetic variance among populations, genetic variance within populations, variance due to the genotype ×temperature interaction, and different responses of genotype to concentration effects, respectively (Qin et al., 2016; Zhan & McDonald, 2011).

Population differentiation in azoxystrobin tolerance (*Q*_ST_) was estimated using the formula described previously (Spitze, 1993; Yang et al., 2016; Zhan & McDonald, 2011). Population differentiation for SSR marker loci was estimated by the fixation index *F*_ST_ (Meirmans & Hedrick, 2011), using POPGENE (Yeh et al., 2000). Statistical difference between overall *F*_ST_ and combined *Q*_ST_ was tested using the SD of *F*_ST_ constructed from 100 bootstraps of the original data.

The heritability and plasticity of azoxystrobin tolerance were estimated separately for each experimental temperature. Heritability was calculated by dividing the genetic variance within populations, that is, I(P), by the total phenotypic variance, and plasticity was measured by dividing the variance of the genotype × concentration interaction, that is, I(P)*C, by the total phenotypic variance (Falconer et al., 1996; Sambandan et al., 2008; Tonsor et al., 2013). Monthly temperature presented as an average over 15–30 years for each collection site was downloaded from World Climate (http://www.worldclimate.com/). Annual mean temperature was estimated by taking the mean of the temperatures between January and December. Pearson's correlation (Lawrence & Lin, 1989) was used to evaluate the association among parameters.

## 3 RESULTS

**FIGURE 1:**
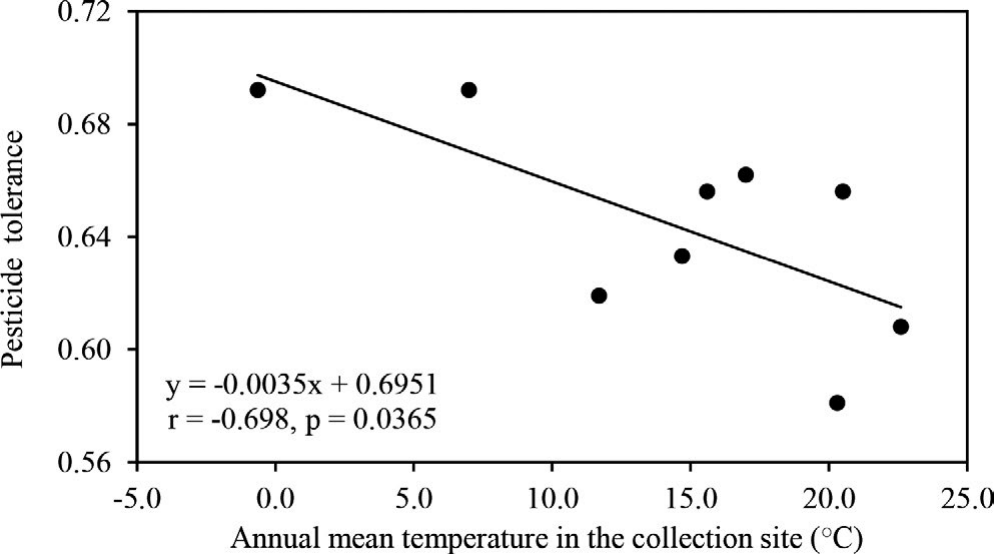
Correlation between azoxystrobin tolerance of *Phytophthora infestans* populations and average local temperature at the collection site. The tolerance, measured by the RGR of isolates in the presence to the absence of the fungicide, was estimated by taking the average across all isolates in each population under the three azoxystrobin concentrations. (0.05, 0.10, and 0.30 μg/ml)

Analysis of variance indicated significant contributions of collection site (population), genotype (isolate), experimental temperature, and fungicide concentration to the level of azoxystrobin tolerance in *P*.* infestans* (*p* < 0.001). The level of azoxystrobin tolerance in the *P*.* infestans* populations was also influenced by the interaction of experimental temperature with pathogen's genotype and collection site (*p* < 0.0001). Overall, *P*.* infestans* populations sampled from cold regions (Inner Mongolia and Ningxia) showed the highest tolerance to azoxystrobin, while the pathogen populations from warm regions (Xiapu and Guangxi but not Fuzhou) showed the least tolerance to azoxystrobin (Table 1). The pathogen populations from temperate regions (Yunnan and Hubei) displayed an intermediate level of tolerance. Further analysis revealed that azoxystrobin tolerance in the nine *P*.* infestans* populations was negatively correlated with the annual mean temperature of collection sites (Figure 1).

**TABLE 1:** Azoxystrobin tolerance measured by the relative growth rate in the presence to the absence of the fungicide in the nine *Phytophthora infestans* populations at the five experimental temperatures

Population	13°C	16°C	19°C	22°C	25°C	Mean
Inner Mongolia	0.543^B^	0.776^A^	0.761^AB^	0.787^A^	0.591^B^	0.692^A^
Ningxia	0.534^B^	0.739^B^	0.763^A^	0.711^CD^	0.709^A^	0.692^A^
Gansu	0.557^B^	0.706^C^	0.662^D^	0.651^E^	0.521^C^	0.619^D^
Guizhou	0.530^B^	0.663^E^	0.769^A^	0.625^E^	0.580^B^	0.633^C^
Yunnan	0.586^A^	0.688^D^	0.762^AB^	0.649^E^	0.595^B^	0.656^B^
Hubei	0.588^A^	0.710^C^	0.743^B^	0.686^D^	0.581^B^	0.662^B^
Xiapu	0.541^B^	0.664^E^	0.705^C^	0.584^F^	0.413^D^	0.581^E^
Fuzhou	0.532^B^	0.689^D^	0.752^AB^	0.745^B^	0.564^B^	0.656^B^
Guangxi	0.519^B^	0.660^E^	0.698^C^	0.731^BC^	0.433^D^	0.608^D^
Average	0.548	0.699	0.735	0.685	0.554	0.644
SD	0.025	0.039	0.038	0.064	0.090	0.051

Note

Values followed by different letters in the same column differ significantly at *p* = 0.05.

Abbreviation: SD, Standard deviation.

**FIGURE 2:**
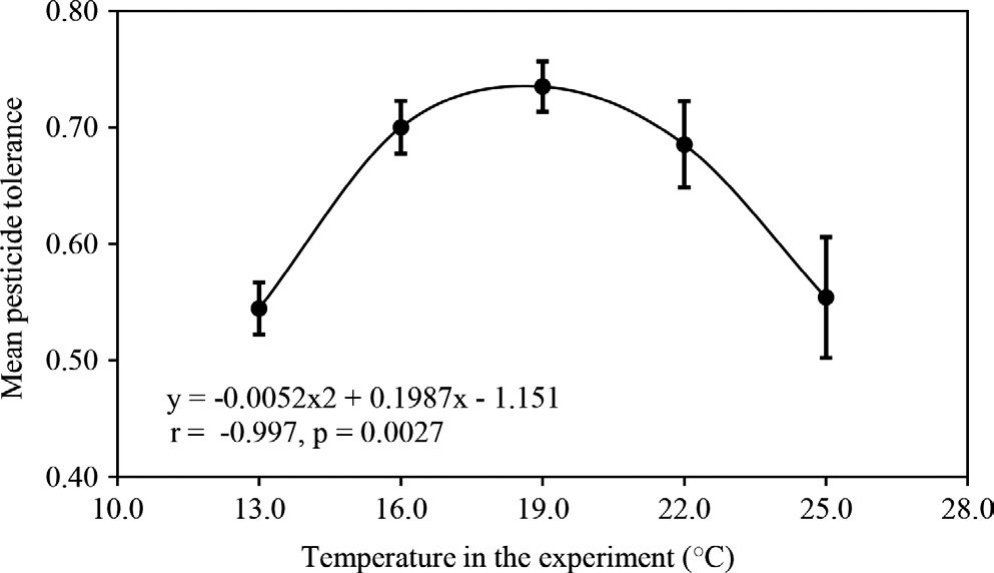
Thermal reaction norm of *Phytophthora infestans* tolerance to azoxystrobin under five experimental temperatures. The tolerance, measured by the RGR of isolates in the presence to the absence of the fungicide, and its error bar (95% confidence intervals) were computed across all isolates in each population under the three azoxystrobin concentrations. (0.05, 0.10, and 0.30 μg/ml)

**FIGURE 3:**
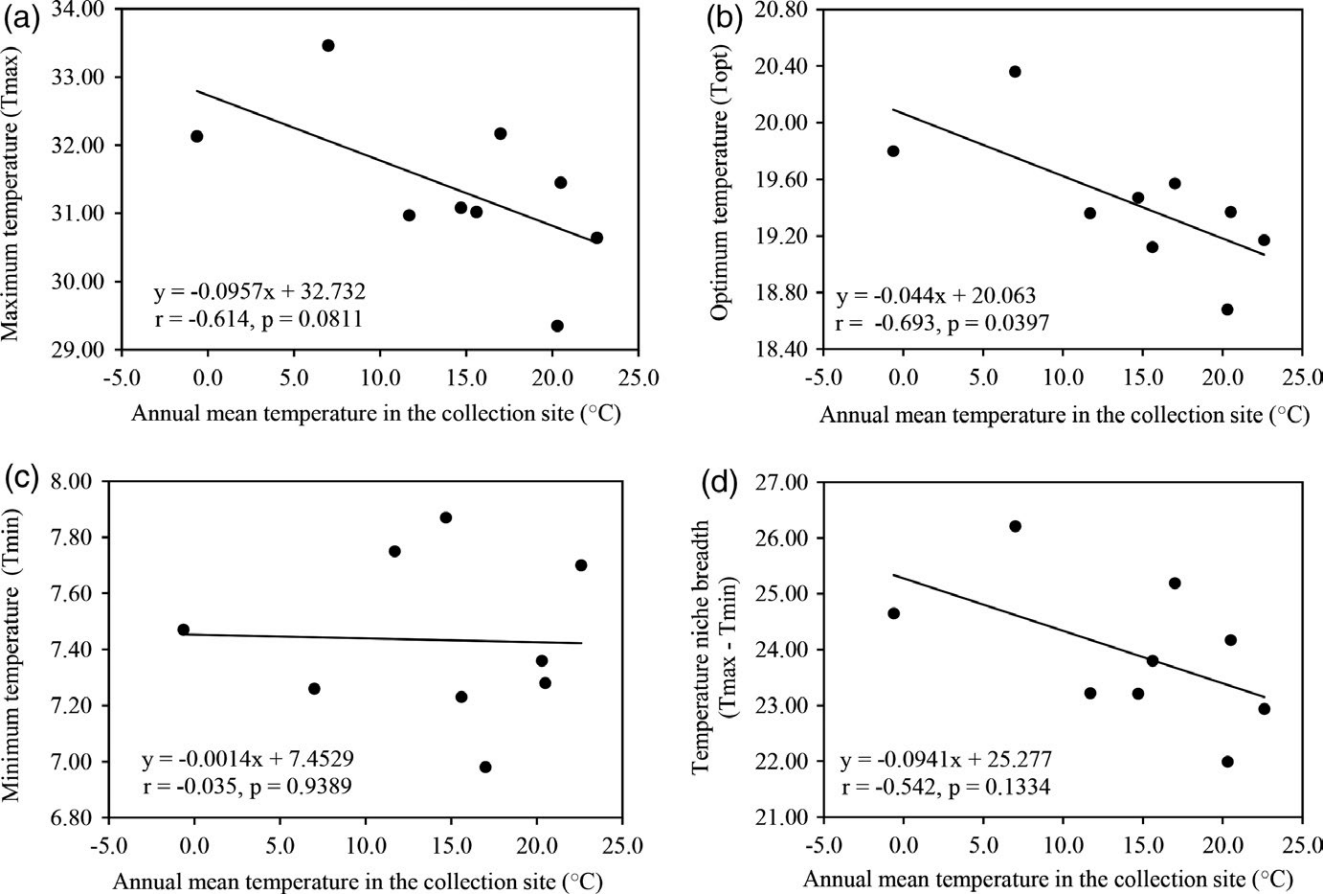
Impact of annual mean temperature at the collection sites on the mean (estimated from 0.05, 0.10, and 0.30 μg/ml fungicide concentrations) adaptive temperatures of *Phytophthora infestans* populations to azoxystrobin: (a) maximum temperature (*T*_max_); (b) optimum temperature (*T*_opt_); (c) minimum temperature (*T*_min_); and (d) temperature niche breadth (*T*_max_ ‐ *T*_min_)

**FIGURE 4:**
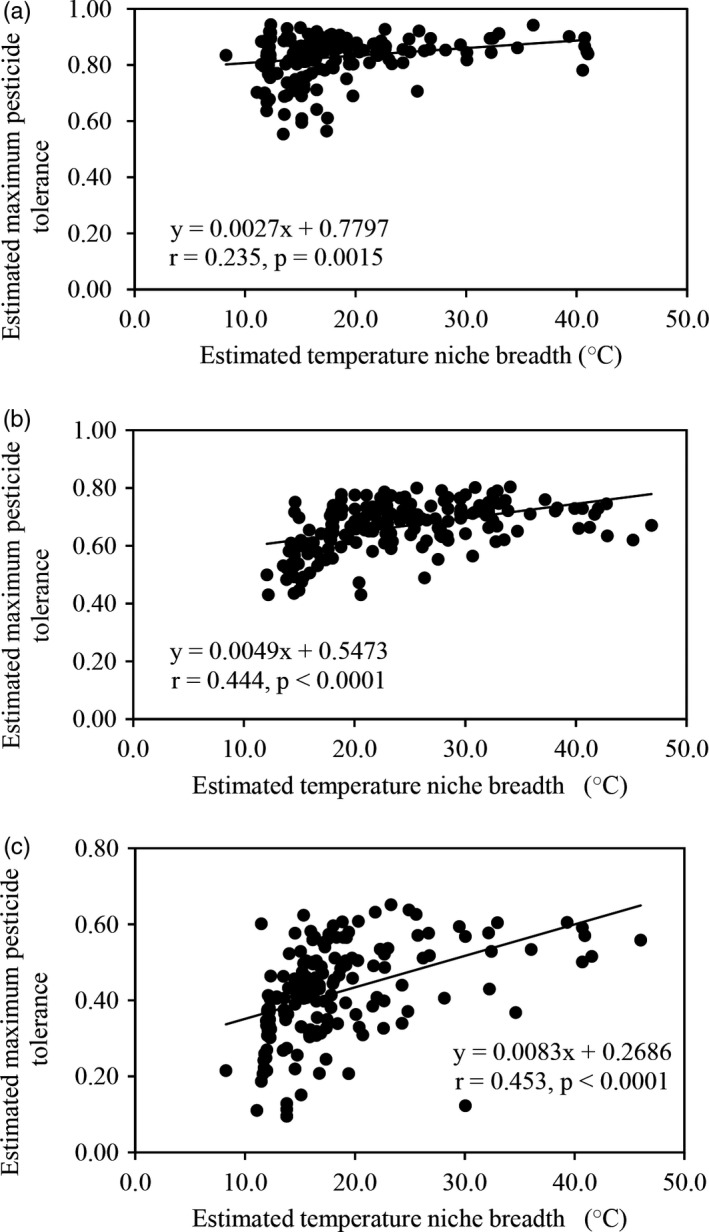
Correlation between the estimated maximum fungicide tolerance of the 180 *Phytophthora infestans* isolates and their estimated temperature niche breadth (*T*_max_ ‐ *T*_min_) at the three azoxystrobin concentrations: (a) 0.05 μg/ml azoxystrobin; (b) 0.10 μg/ml azoxystrobin; and (c) 0.30 μg/ml azoxystrobin

Thermal reaction norm, the profile of *P*.* infestans* tolerance to azoxystrobin in response to the change of experimental temperature, showed a good fit to a second order polynomial (*r* = 0.997, *p* = 0.0027, Figure 2), and variance in azoxystrobin tolerance among the 180 *P*.* infestans* isolates meets the assumption of homoscedasticity under all five experimental temperatures (data not shown). The estimated maximum (*T*_max_), optimum (*T*_opt_), minimum (*T*_min_) temperatures, and temperature niche breadth (*T*_max_ ‐ *T*_min_) of the fungicide tolerance varied significantly among the pathogen populations (Table 2) and were negatively associated with the annual mean temperature of collection sites although only one correlation (i.e., *T*_opt_) was significant (Figure 3). The optimum temperature for azoxystrobin tolerance in *P*.* infestans* was ~19°C although variation (~ 2°C) existed among populations sampled from different locations (Table 2). The temperature niche breadth (*T*_max_‐ *T*_min_) of azoxystrobin tolerance reduced as the fungicide concentration increased, changing from 29.61°C in 0.05 μg/ml to 24.16°C in 0.10 μg/ml and then 18.02°C in 0.30 μg/ml (Table 2) and was positively associated with the maximum fungicide tolerance of the pathogen at all three concentrations (Figure 4).

**TABLE 2:** Duncan's multiple range tests for differences in the estimated maximum (*T*_max_), optimum (*T*_opt_.) and minimum (*T*_min_) temperatures (°C) of azoxystrobin tolerance in the *Phytophthora infestans* populations under the three azoxystrobin concentrations sampled (arranged from lowest mean annual temperature at the top to highest mean annual temperature at the bottom)

Population	0.05 μg/ml			0.10 μg/ml			0.30 μg/ml		
	*T*_max_	*T*_opt_	*T*_min_	*T*_max_	*T*_opt_	*T*_min_	*T*_max_	*T*_opt_	*T*_min_
Inner Mongolia	32.44^D^	18.54^D^	4.65^AB^	33.92^A^	20.62^A^	7.31^B^	30.02^B^	20.24^B^	10.46^AB^
Ningxia	35.28^AB^	19.69^A^	4.11^BC^	33.00^AB^	20.32^AB^	7.65^AB^	32.11^A^	21.06^A^	10.01^B^
Gansu	34.12^BC^	19.13^BC^	4.14^BC^	30.17^C^	19.44^CD^	8.71^A^	28.62^BC^	19.51^BCD^	10.39^AB^
Guizhou	36.01^A^	19.64^AB^	3.27^C^	29.82^C^	19.27^CD^	8.73^A^	27.42^CD^	19.51^BCD^	11.61^A^
Yunnan	32.43^D^	18.77^CD^	5.12^A^	33.08^AB^	19.79^BC^	6.50^B^	27.56^CD^	18.81^D^	10.06^B^
Hubei	34.73^ABC^	19.42^AB^	4.12^BC^	32.92^AB^	19.88^ABC^	6.84^B^	28.86^BC^	19.42^CD^	9.98^B^
Xiapu	29.85^E^	17.55^E^	3.33^C^	29.92^C^	18.79^D^	7.66^AB^	28.28^BCD^	19.69^BC^	11.10^AB^
Fuzhou	35.29^AB^	19.78^A^	4.26^ABC^	31.31^BC^	19.38^CD^	7.46^B^	27.76^CD^	18.94^CD^	10.13^B^
Guangxi	33.49^CD^	18.78^CD^	4.06^BC^	31.84^ABC^	19.78^BC^	7.73^ABC^	26.59^D^	18.94^CD^	11.30^AB^
Average	33.73	19.03	4.12	31.78	19.70	7.62	28.58	19.57	10.56

Note

Values followed by different letters in a column are significantly different from each other at *p* = 0.05 (as the fungicide concentration increases, the maximum range of temperature for growth decreases while the minimum temperature for growth increases).

**FIGURE 5:**
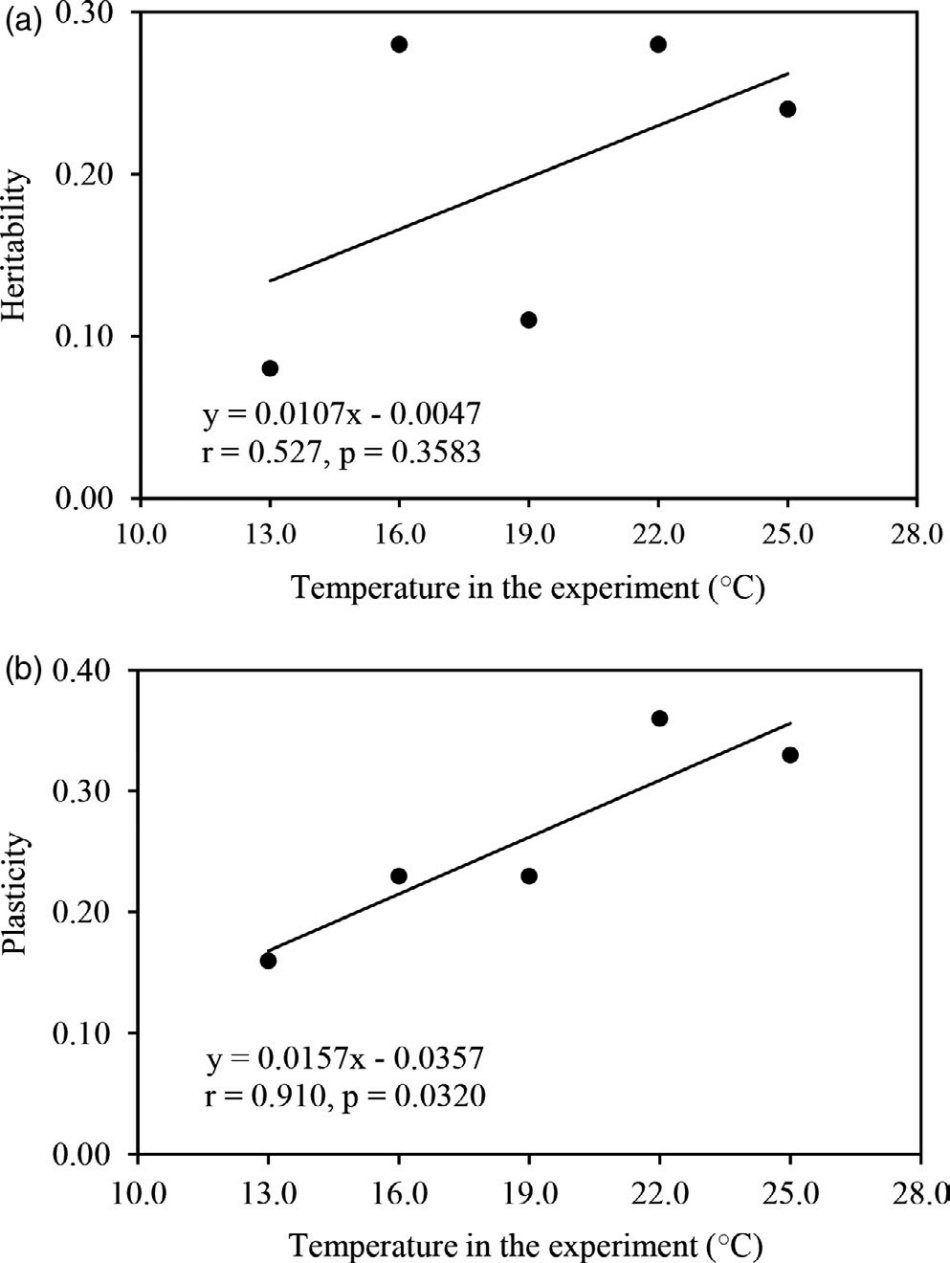
The impact of experimental temperatures on the estimate of within‐population variation in azoxystrobin tolerance: (a) Heritability and (b) Plasticity. The heritability and plasticity were estimated by using the mean azoxystrobin tolerance of *P*.* infestans* isolates across three fungicide concentrations (0.05, 0.10, and 0.30 μg/ml)

Genetic variance in azoxystrobin tolerance (heritability) accounted for 8%–28% (mean 20%) of the phenotypic variation while the variance of the genotype–environment interaction (phenotypic plasticity) accounted for 16%–36% (mean 26%) of the phenotypic variation (Table 3). The ratio between phenotypic plasticity and heritability in azoxystrobin tolerance ranged from 0.82 at 16°C to 2.09 at 19°C with an average of 1.51. Phenotypic plasticity in azoxystrobin tolerance was positively and significantly associated with experimental temperature (Figure 5b). Though heritability in azoxystrobin tolerance was also positively correlated with experimental temperature, the association was not statistically significant (Figure 5a).

**TABLE 3:** Heritability and phenotypic plasticity in azoxystrobin tolerance

Temperature	Plasticity (P)	Heritability (H)	P:H
13°C	0.16	0.08	2.00
16°C	0.23	0.28	0.82
19°C	0.23	0.11	2.09
22°C	0.36	0.28	1.29
25°C	0.33	0.24	1.38
Average	0.26	0.20	1.51

Note

The tolerance was measured by relative growth rate in the presence to the absence of fungicide, and its heritability and plasticity were estimated by using the mean azoxystrobin tolerance of the *P*.* infestans* isolates over three fungicide concentrations (0.05, 0.10, and 0.30 μg/ml).

**FIGURE 6:**
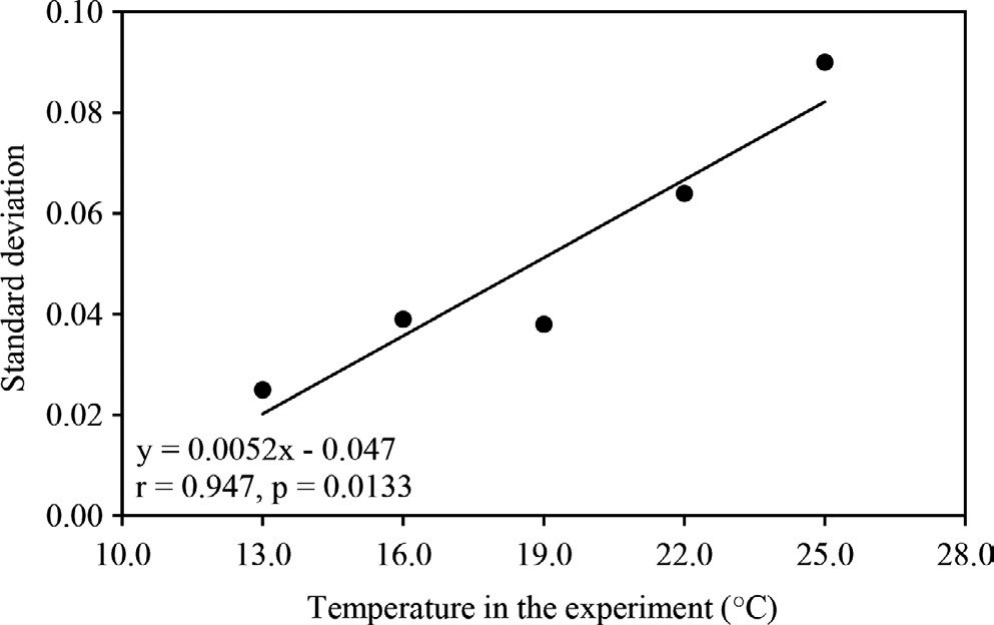
Variation (SD) of azoxystrobin tolerance among *P*.* infestans* populations sampled from different geographic locations in response to the five temperatures used in the experiment. The SD was estimated from mean tolerance of each isolate over three azoxystrobin concentrations (0.05, 0.10, and 0.30 μg/ml)

The variation in population tolerance of *P*.* infestans* to azoxystrobin measured by SD (Table 1) was also positively and significantly associated with experimental temperature (Figure 6). The overall population differentiation (*Q*_ST_) in azoxystrobin tolerance across fungicide concentrations and experimental temperatures was 0.24, which was significantly higher than 0.16, the overall population differentiation (*F*_ST_) in SSR marker loci (*p* < 0.0001).

## 4 DISCUSSION

In this study, the temperature‐mediated evolution of pesticide tolerance was investigated in the Irish famine pathogen *P*.* infestans* using a common garden approach (Yang et al., 2016; Zhan & McDonald, 2011). Results reveal that thermal conditions in the experiments have a significant impact on the azoxystrobin tolerance of the *P*.* infestans* populations (Table 1, Figure 2). This is consistent with previous reports involving other pathogen–pesticide interactions (He et al., 2018; Matthiesen et al., 2016; Rodríguez‐Verdugo et al., 2013; Zhang et al., 2015). More importantly, for the first time, we demonstrate that experimental temperature can strongly affect the spatial distribution of pesticide tolerance across pathogen populations. As temperature in the experiments increased, spatial differentiation in azoxystrobin tolerance among the *P*.* infestans* populations sampled from different geographic regions also increased (Figure 6, *r* = 0.947, *p* = 0.0133). These associations indicate that air temperature greatly affects the level and spatial variation of azoxystrobin efficacy, posing a challenge for a broad recommendation to chemically control plant diseases under future temperature scenarios (IPCC, 2014). Under this scenario, any preventive or therapeutic strategies (e.g., pesticide types or doses) which are suitable for one geographic location or temperature may not be adequate for another location or temperature.

Statistical analysis reveals that *P*.* infestans* populations varied significantly in azoxystrobin tolerance under all five experimental temperatures (Table 1). Both nonadaptive differentiation caused by a stochastic event [random genetic drift, (Stefansson et al., 2014)] and adaptive differentiation caused by a determinant event (natural selection, (Olson‐Manning et al., 2012)) can lead to the tolerance variation, but the two events can be separated by a comparative analysis of population differentiations in neutral genomes (*F*_ST_) and azoxystrobin tolerance (*Q*_ST_) as described in previous publications (Qin et al., 2016; Yang et al., 2016). Stochastic events are expected to affect the entire genome equally, resulting in similar population differentiation in neutral genome and azoxystrobin tolerance. On the other hand, a determinant event should only affect sequences directly or indirectly involving biological and ecological functions, leading to a significant difference in population differentiation in *Q*_ST_ and *F*_ST_. When this comparative analysis was conducted in the study, we found that natural selection for genotypes adapting to local environments was responsible for the difference in azoxystrobin tolerance among the *P*.* infestans* populations sampled from different locations as indicated by a significantly higher *Q*_ST_ than *F*_ST_. Although other possibilities cannot be completely excluded, local air temperature is one of the drivers for the spatial differentiation as shown by the strong association between annual mean temperature at the collection site and azoxystrobin tolerance in the pathogen (Figure 1). Our study also shows positive associations of heritability and plasticity in azoxystrobin tolerance with experimental temperature (Figure 5), indicating that the two genetic parameters increase in parallel to elevating environmental temperature although the relationship involving heritability was not significant. Heritability, measured by the proportion of additive genetic variance to phenotypic variance of traits (Andrew et al., 2010), is generated by permanent changes of genetic material in genomes, while plasticity is a phenomenon whereby the same genotype produces different phenotypes through alternation of gene expression or enzymatic activity in response to environmental fluctuations (Draghi & Whitlock, 2012). Both heritability and plasticity are heritable (Pelletier et al., 2007) and fundamental to the ability of traits to evolve. Fisher's fundamental theorem of natural selection hypotheses that the ability of adaptation to changing environments is governed by genetic variance in heritable traits that is relevant to fitness (Fisher, 1930). Species with higher genetic variation are expected to have greater evolutionary potential and adapt more quickly to environmental stresses. Taken together, this result suggests that global warming may enhance the risk of pathogens developing pesticide resistance, primarily through alternation of gene expression or enzymatic activity (plasticity), and further highlights the challenge of administrating pesticides to effectively control plant diseases for agricultural production and ecological sustainability under future climatic conditions (Laetz et al., 2014; Nørhave et al., 2012).

Models predict that global warming is likely to be accompanied by an increased occurrence of extreme temperatures in both directions (Mann et al., 2018; Rahmstorf & Coumou, 2011). This pattern of thermal fluctuations is expected to select for pathogen phenotypes with broader temperature niches. It is, therefore, worrying to find that pathogen genotypes with broader temperature niches also tend to have a higher level of tolerance to pesticides as demonstrated by the positive association between azoxystrobin tolerance and temperature niche breadth in *P*.* infestans* (Figure 4). This raises further concerns that the erosion of pesticide efficacy may be escalated under global warming (Chan et al., 2018; Shuman, 2011).

A previous publication (Yang et al., 2016) demonstrated that the intrinsic growth rate of *P*.* infestans* in the absence of pesticides showed a bell‐shaped distribution, initially increasing from 13°C, reaching a peak at 19°C, and then declining gradually as temperature rose further. Because azoxystrobin tolerance of *P*.* infestans* in the current study was derived by the ratio of the intrinsic growth rate of the pathogen isolates in the presence to the absence of the pesticide, it is expected that a mirror distribution (U‐shaped) will be seen in the level of azoxystrobin tolerance, showing lowest tolerance at the optimum temperature for *P*.* infestans* growth (~19°C). Such a U‐shaped distribution was found in the toxicity of deltamethrin and bendiocarb, two pesticides used to eradicate malaria vectors *Anopheles arabiensis* and *Anopheles funestus*. In those interactions, toxicity reduced at both lower and higher than normal temperature (Glunt et al., 2018). Surprisingly, we found that azoxystrobin tolerance in *P*.* infestans* failed to follow this pattern; rather, it also followed a bell‐shaped distribution (Figure 2). In addition to mutation in target genes (Holmes et al., 2016), pesticide tolerance can be achieved by many detoxifying processes in pathogens such as oxidation, reduction, hydrolysis, hydration, conjugation, condensation, and isomerization (Yang et al., 2013). All of these processes are associated with metabolic rate (Auer et al., 2018; Clarke, 2006). It is reasonable to believe that accelerated metabolism in cells at their optimum growth temperature facilitates these detoxifying processes, leading to the greatest pesticide tolerance at this thermal point. Pesticide tolerance through these detoxifying processes is likely to be nonspecific and can be adopted by pathogens to battle against a wide spectrum of artificial and natural substances.

In conclusion, our results indicate that air temperature plays an important role in the evolution of pesticide tolerance in pathogens. Global warming is expected to escalate evolutionary potential of pesticide tolerance in pathogens and to polarize its spatial distribution, increasing the difficulty of managing the plant and human diseases effectively for agricultural production and ecological sustainability in future climatic conditions. Previous studies found that *P*.* infestans* isolates with a same clonal lineage varied morphologically and functionally including pesticide tolerance (Saville et al., 2014; Schepers et al., 2018) and temperature sensitivity (Cooke et al., 2012; Mariette et al., 2016). In our study, some of multilocus genotypes included in the experiment only differ in 1–2 loci and, therefore, could belong to the same clonal lineage. Regardless, this possibility would not affect our conclusions due to: (i) clonal lineages are more often found among isolates within a location than among locations and (ii) the same set of multilocus genotypes were used to measure the influences of experimental temperature on azoxystrobin tolerance.

## CONFLICT OF INTEREST

The authors declared no any conflict of interest.

## Supporting information

Table S1

## Data Availability

Data of geographic coordinate and annual mean temperature (mean, variance, and SD) of the nine populations sampled for *Phytophthora infestans* used in this study are presented in Table S1. Other associated data will be deposited in Dryad when the manuscript is accepted for publication.

## References

[eva13197-bib-0001] Aguayo, J. , Elegbede, F. , Husson, C. , Saintonge, F. X. , Marçais, B. (2014). Modeling climate impact on an emerging disease, the Phytophthora alni‐induced alder decline. Global Change Biology, 20(10), 3209–3221. 10.1111/gcb.12601 24729529

[eva13197-bib-0002] Andrew, R. L. , Wallis, I. R. , Harwood, C. E. , & Foley, W. J. (2010). Genetic and environmental contributions to variation and population divergence in a broad‐spectrum foliar defence of *Eucalyptus tricarpa* . Annals of Botany, 105(5), 707–717. 10.1093/aob/mcq034 20228089 PMC2859910

[eva13197-bib-0003] Auer, S. K. , Dick, C. A. , Metcalfe, N. B. , & Reznick, D. N. (2018). Metabolic rate evolves rapidly and in parallel with the pace of life history. Nature Communications, 9(1), 14. 10.1038/s41467-017-02514-z PMC5750215 29295982

[eva13197-bib-0004] Avenot, H. , Sellam, A. , & Michailides, T. (2009). Characterization of mutations in the membrane‐anchored subunits AaSDHC and AaSDHD of succinate dehydrogenase from *Alternaria alternata* isolates conferring field resistance to the fungicide boscalid. Plant Pathology, 58(6), 1134–1143. 10.1111/j.1365-3059.2009.02154.x

[eva13197-bib-0005] Bartlett, D. W. , Clough, J. M. , Godwin, J. R. , Hall, A. A. , Hamer, M. , & Parr‐Dobrzanski, B. (2002). The strobilurin fungicides. Pest Management Science, 58(7), 649–662. 10.1002/ps.520 12146165

[eva13197-bib-0006] Birch, P. R. , Bryan, G. , Fenton, B. , Gilroy, E. M. , Hein, I. , Jones, J. T. , Prashar, A. , Taylor, M. A. , Torrance, L. & Toth, I. K. (2012). Crops that feed the world 8: Potato: Are the trends of increased global production sustainable? Food Security, 4(4), 477–508. 10.1007/s12571-012-0220-1

[eva13197-bib-0007] Brunner, P. C. , Stefansson, T. S. , Fountaine, J. , Richina, V. , & McDonald, B. (2016). A global analysis of CYP51 diversity and azole sensitivity in *Rhynchosporium commune* . Phytopathology, 106(4), 355–361. 10.1094/PHYTO-07-15-0158-R 26623995

[eva13197-bib-0008] Chan, A. , Hon, K. , Leung, T. , Ho, M. , Rosa Duque, J. , & Lee, T. (2018). The effects of global warming on allergic diseases. Hong Kong Medical Journal, 24(3), 277–284. 10.12809/hkmj177046 29808822

[eva13197-bib-0009] Chen, C.‐J. , Yu, J.‐J. , Bi, C.‐W. , Zhang, Y.‐N. , Xu, J.‐Q. , Wang, J.‐X. , & Zhou, M.‐G. (2009). Mutations in a β‐tubulin confer resistance of Gibberella zeae to benzimidazole fungicides. Phytopathology, 99(12), 1403–1411. 10.1094/phyto-99-12-1403 19900007

[eva13197-bib-0010] Chen, F. , Duan, G. H. , Li, D. L. , & Zhan, J. (2017). Host resistance and temperature‐dependent evolution of aggressiveness in the plant pathogen *Zymoseptoria tritici* . Frontier Microbiology, 8, 1217. 10.3389/fmicb.2017.01217 PMC5487519 28702023

[eva13197-bib-0011] Chen, F. , Liu, X. , & Schnabel, G. (2013). Field strains of *Monilinia fructicola* resistant to both MBC and DMI fungicides isolated from stone fruit orchards in the eastern United States. Plant Disease, 97(8), 1063–1068. 10.1094/pdis-12-12-1177-re 30722486

[eva13197-bib-0012] Chen, F. , Zhou, Q. , Qin, C. , Li, Y. , & Zhan, J. (2018). Low evolutionary risk of iprovalicarb resistance in *Phytophthora infestans* . Pesticide Biochemistry and Physiology, 152, 76–83. 10.1016/j.pestbp.2018.09.003 30497714

[eva13197-bib-0013] Chen, F. , Zhou, Q. , Xi, J. , Li, D. L. , Schnabel, G. , & Zhan, J. (2018). Analysis of RPA190 revealed multiple positively selected mutations associated with metalaxyl resistance in *Phytophthora infestans* . Pest Management Science, 74, 1916–1924. 10.1002/ps.4893 29457681

[eva13197-bib-0014] Clarke, A. (2003). Costs and consequences of evolutionary temperature adaptation. Trends in Ecology and Evolution, 18(11), 573–581. 10.1016/j.tree.2003.08.007

[eva13197-bib-0015] Clarke, A. (2006). Temperature and the metabolic theory of ecology. Functional Ecology, 20(2), 405–412. 10.1111/j.1365-2435.2006.01109.x

[eva13197-bib-0016] Cooke, D. E. , Cano, L. M. , Raffaele, S. , Bain, R. A. , Cooke, L. R. , Etherington, G. J. , Deahl, K. L. , Farrer, R. A. , Gilroy, E. M. , Goss, E. M. , Grünwald, N. J. , Hein, I. , MacLean, D. , McNicol, J. W. , Randall, E. , Oliva, R. F. , Pel, M. A. , Shaw, D. S. , Squires, J. N. , … Kamoun, S. (2012). Genome analyses of an aggressive and invasive lineage of the Irish potato famine pathogen. PLOS Pathogen, 8(10): e1002940. 10.1371/journal.ppat.1002940 23055926 PMC3464212

[eva13197-bib-0017] Cuco, A. P. , Castro, B. B. , Gonçalves, F. , Wolinska, J. , & Abrantes, N. (2018). Temperature modulates the interaction between fungicide pollution and disease: Evidence from a Daphnia‐microparasitic yeast model. Parasitology, 145(7), 939–947. 10.1017/s0031182017002062. 29160185

[eva13197-bib-0018] Danies, G. , Myers, K. , Mideros, M. F. , Restrepo, S. , Martin, F. N. , Cooke, D. E. L. , Smart, C. D. , Ristaino, J. B. , Seaman, A. J. , Gugino, B. K. , Grünwald, N. J. , & Fry, W. E. (2014). An ephemeral sexual population of *Phytophthora infestans* in the northeastern United States and Canada. PLoS One, 9(12), e116354. 10.1371/journal.pone.0116354 25551215 PMC4281225

[eva13197-bib-0019] Delnat, V. , Tran, T. T. , Janssens, L. , & Stoks, R. (2019). Daily temperature variation magnifies the toxicity of a mixture consisting of a chemical pesticide and a biopesticide in a vector mosquito. Science of the Total Environment, 659, 33–40. 10.1016/j.scitotenv.2018.12.332 30594859

[eva13197-bib-0020] Draghi, J. A. , & Whitlock, M. C. (2012). Phenotypic plasticity facilitates mutational variance, genetic variance, and evolvability along the major axis of environmental variation. Evolution, 66(9), 2891–2902. 10.1111/j.1558-5646.2012.01649.x 22946810

[eva13197-bib-0021] Du, B. , Zhang, Z. , Liu, W. , Ye, Y. , Lu, T. , Zhou, Z. , Li, Y. , Fu, Z. , & Qian, H. (2019). Acute toxicity of the fungicide azoxystrobin on the diatom *Phaeodactylum tricornutum* . Ecotoxicology and Environmental Safety, 168, 72–79. 10.1016/j.ecoenv.2018.10.074 30384169

[eva13197-bib-0022] Falconer, D. , Mackay, T. , & Bulmer, M. (1996). Introduction to quantitative genetics. American Journal of Human Genetics, 68(2), 183.

[eva13197-bib-0023] Fisher, R. A. (1930). The genetical theory ofnatural selection. Oxford: Clarendon Press. 10.5962/bhl.title.27468

[eva13197-bib-0024] Flier, W. G. , Kroon, L. P. N. M. , Hermansen, A. , van Raaij, H. M. G. , Speiser, B. , Tamm, L. , Fuchs, J. G. , Lambion, J. , Razzaghian, J. , Andrivon, D. , Wilcockson, S. , & Leifert, C. (2007). Genetic structure and pathogenicity of populations of *Phytophthora infestans* from organic potato crops in France, Norway, Switzerland and the United Kingdom. Plant Pathology, 56(4), 562–572. 10.1111/j.1365-3059.2007.01571.x

[eva13197-bib-0025] FRAC (2012). List of pathogens with field resistance towards QoI fungicides. Fungicide Resistance Action Committee’s 2012. Retrieved from: http://www.frac.info/docs/default‐source/qoi‐wg/qoi‐quickreferences/species‐with‐qo‐resistance

[eva13197-bib-0026] Fry, W. (2008). *Phytophthora infestans*: The plant (and R gene) destroyer. Molecular Plant Pathology, 9(3), 385–402. 10.1111/j.1364-3703.2007.00465.x 18705878 PMC6640234

[eva13197-bib-0027] Gisi, U. , & Sierotzki, H. (2008). Fungicide modes of action and resistance in downy mildews. The Downy Mildews‐Genetics Molecular Biology and Control, 122(1), 157–167. 10.1007/s10658-008-9290-5

[eva13197-bib-0028] Glunt, K. D. , Oliver, S. V. , Hunt, R. H. , & Paaijmans, K. P. (2018). The impact of temperature on insecticide toxicity against the malaria vectors *Anopheles arabiensis* and *Anopheles funestus* . Malaria Journal, 17(1), 131. 10.1186/s12936-018-2250-4 29606123 PMC5879579

[eva13197-bib-0029] Guo, L. , Zhu, X.‐Q. , Hu, C.‐H. , & Ristaino, J. B. (2010). Genetic structure of *Phytophthora infestans* populations in China indicates multiple migration events. Phytopathology, 100(10), 997–1006. 10.1094/phyto-05-09-0126 20839935

[eva13197-bib-0030] Han, M. , Liu, G. , Li, J. P. , Govers, F. , Zhu, X. Q. , Shen, C. Y. , & Guo, L. Y. (2013). *Phytophthora infestans* field isolates from Gansu Province, China are genetically highly diverse and show a high frequency of self fertility. Journal of Eukaryotic Microbiology, 60(1), 79–88. 10.1111/jeu.12010 23194320

[eva13197-bib-0031] Haverkort, A. J. A. S. (1990). Ecology of potato cropping systems in relation to latitude and altitude. Agricultural Systems, 32(3), 251–272. 10.1016/0308-521X(90)90004-A

[eva13197-bib-0032] He, M.‐H. , Li, D.‐L. , Zhu, W. , Wu, E.‐J. , Yang, L.‐N. , Wang, Y.‐P. , Waheed, A. , & Zhan, J. (2018). Slow and temperature‐mediated pathogen adaptation to a nonspecific fungicide in agricultural ecosystem. Evolutionary Applications, 11(2), 182–192. 10.1111/eva.12526 29387154 PMC5775493

[eva13197-bib-0033] Heaney, S. , Hall, A. , Davies, S. , & Olaya, G. (2000). Resistance to fungicides in the Qol‐STAR cross‐resistance group: Current perspectives. Paper presented at the The BCPC Conference: Pests and diseases, Volume 2. Proceedings of an international conference held at the Brighton Hilton Metropole Hotel, Brighton, UK, 13–16 November 2000 (pp. 755–762). British Crop Protection Council,.

[eva13197-bib-0034] Hoffmann, A. A. , & Sgro, C. M. (2011). Climate change and evolutionary adaptation. Nature, 470(7335), 479–485. 10.1038/nature09670 21350480

[eva13197-bib-0035] Holmes, A. H. , Moore, L. S. , Sundsfjord, A. , Steinbakk, M. , Regmi, S. , Karkey, A. , Guerin, P. J. , & Piddock, L. J. (2016). Understanding the mechanisms and drivers of antimicrobial resistance. The Lancet, 387(10014), 176–187. 10.1016/S0140-6736(15)00473-0 26603922

[eva13197-bib-0036] Hwang, Y. T. , Wijekoon, C. , Kalischuk, M. , Johnson, D. , Howard, R. , Prüfer, D. , & Kawchuk, L. (2014). Evolution and management of the Irish potato famine pathogen *Phytophthora infestans* in Canada and the United States. American Journal of Potato Research, 91(6), 579–593. 10.1007/s12230-014-9401-0

[eva13197-bib-0037] IPCC (2014). Climate change 2014: Synthesis report. In Core writing team , R. K. Pachauri , & L. A. Meyer (Eds.). Contribution of Working Groups I. II and III to the Fifth Assessment Report of the Intergovernmental Panel on Climate Change (pp. 151). Geneva, Switzerland: IPCC.

[eva13197-bib-0038] Jinks, J. L. , & Grindle, M. (1963). Changes induced by training in *Phytophthora infestans* . Heredity, 18, 245–264.

[eva13197-bib-0039] Kessel, G. J. , Mullins, E. , Evenhuis, A. , Stellingwerf, J. , Cortes, V. O. , Phelan, S. , van den Bosch, T. , Förch, M. G. , Goedhart, P. & van der Voet, H. (2018). Development and validation of IPM strategies for the cultivation of cisgenically modified late blight resistant potato. European Journal of Agronomy, 96, 146–155. 10.1016/j.eja.2018.012

[eva13197-bib-0040] Knapova, G. , & Gisi, U. (2002). Phenotypic and genotypic structure of *Phytophthora infestans* populations on potato and tomato in France and Switzerland. Plant Pathology, 51(5), 641–653. 10.1046/j.1365-3059.2002.00750.x

[eva13197-bib-0041] Knies, J. L. , Izem, R. , Supler, K. L. , Kingsolver, J. G. , & Burch, C. L. (2006). The genetic basis of thermal reaction norm evolution in lab and natural phage populations. PLoS Biology, 4(7), e201. 10.1371/journal.pbio.0040201 16732695 PMC1472247

[eva13197-bib-0042] Kokalis‐Burelle, N. , Butler, D. M. , & Rosskopf, E. N. (2013). Evaluation of cover crops with potential for use in anaerobic soil disinfestation (ASD) for susceptibility to three species of *Meloidogyne* . Journal of Nematology, 45(4), 272. 24379486 PMC3873904

[eva13197-bib-0043] Laetz, C. A. , Baldwin, D. H. , Hebert, V. R. , Stark, J. D. , & Scholz, N. L. (2014). Elevated temperatures increase the toxicity of pesticide mixtures to juvenile coho salmon. Aquatic Toxicology, 146, 38–44. 10.1016/j.aquatox.2013.10.022 24270668

[eva13197-bib-0044] Lamari, L. (2002). *Assess:* Image analysis software for plant disease quantification. St. Paul, Minnesota: APS press.

[eva13197-bib-0045] Lawrence, I. , & Lin, K. (1989). A concordance correlation coefficient to evaluate reproducibility. Biometrics, 45(1), 255–268. 10.2307/2532051 2720055

[eva13197-bib-0046] Lees, A. , Wattier, R. , Shaw, D. , Sullivan, L. , Williams, N. , & Cooke, D. (2006). Novel microsatellite markers for the analysis of *Phytophthora infestans* populations. Plant Pathology, 55(3), 311–319. 10.1111/j.1365-3059.2006.01359.x

[eva13197-bib-0047] Loehle, C. , Idso, C. , & Wigley, T. B. (2016). Physiological and ecological factors influencing recent trends in United States forest health responses to climate change. Forest Ecology and Management, 363, 179–189. 10.1016/j.foreco.2015.12.042

[eva13197-bib-0048] Lucas, J. A. , Hawkins, N. J. , & Fraaije, B. A. (2015). The evolution of fungicide resistance. Advances in Applied Microbiology, 90(1), 29–92. 10.1016/bs.aambs.2014.09.001 25596029

[eva13197-bib-0049] Lurwanu, Y. , Wang, Y. P. , Abdul, W. , Zhan, J. , & Yang, L. N. (2020). Temperature‐mediated plasticity regulates the adaptation of *Phytophthora infestans* to azoxystrobin fungicide. Sustainability, 12(3), 1188. 10.3390/su12031188.

[eva13197-bib-0050] MacFadden, D. R. , McGough, S. F. , Fisman, D. , Santillana, M. , & Brownstein, J. S. (2018). Antibiotic resistance increases with local temperature. Nature Climate Change, 8(6), 510–514, 10.1038/s41558-018-0161-6 PMC6201249 30369964

[eva13197-bib-0051] Maino, J. L. , Umina, P. A. , & Hoffmann, A. A. (2018). Climate contributes to the evolution of pesticide resistance. Global Ecology and Biogeography, 27(2), 223–232. 10.1111/geb.12692

[eva13197-bib-0052] Mann, M. E. , Rahmstorf, S. , Kornhuber, K. , Steinman, B. A. , Miller, S. K. , Petri, S. , & Coumou, D. (2018). Projected changes in persistent extreme summer weather events: The role of quasi‐resonant amplification. Science Advances, 4(10), eaat3272. 10.1126/sciadv.aat3272 30402537 PMC6209391

[eva13197-bib-0053] Mariette, N. , Androdias, A. , Mabon, R. , Corbiere, R. , Marquer, B. , Montarry, J. , & Andrivon, D. (2016). Local adaptation to temperature in populations and clonal lineages of the Irish potato famine pathogen *Phytophthora infestans* . Ecology and Evolution, 6(17), 6320–6331. 10.1002/ece3.2282 27648246 PMC5016652

[eva13197-bib-0054] Matson, M. E. , Small, I. M. , Fry, W. E. , & Judelson, H. S. (2015). Metalaxyl resistance in *Phytophthora infestans*: Assessing role of RPA190 gene and diversity within clonal lineages. Phytopathology, 105(12), 1594–1600. 10.1094/PHYTO-05-15-0129-R 26551315

[eva13197-bib-0055] Matthiesen, R. , Ahmad, A. , & Robertson, A. (2016). Temperature affects aggressiveness and fungicide sensitivity of four *Pythium* spp. that cause soybean and corn damping off in Iowa. Plant Disease, 100(3), 583–591. 10.1094/PDIS-04-15-0487-RE 30688593

[eva13197-bib-0056] Mayton, H. , Smart, C. , Moravec, B. , Mizubuti, E. , Muldoon, A. , & Fry, W. (2000). Oospore survival and pathogenicity of single oospore recombinant progeny from a cross involving US‐17 and US‐8 genotypes of *Phytophthora infestans* . Plant Disease, 84(11), 1190–1196. 10.1094/PDIS.2000.84.11.1190 30832166

[eva13197-bib-0057] Meirmans, P. G. , & Hedrick, P. W. (2011). Assessing population structure: F*_ST_* and related measures. Molecular Ecology Resources, 11(1), 5–18. 10.1111/j.1755-0998.2010.02927.x 21429096

[eva13197-bib-0058] Mohd‐Assaad, N. , McDonald, B. A. , & Croll, D. (2016). Multilocus resistance evolution to azole fungicides in fungal plant pathogen populations. Molecular Ecology, 25(24), 6124–6142. 10.1111/mec.13916 27859799

[eva13197-bib-0059] Nikolaidis, I. , Favini‐Stabile, S. , & Dessen, A. (2014). Resistance to antibiotics targeted to the bacterial cell wall. Protein Science, 23(3), 243–259. 10.1002/pro.2414 24375653 PMC3945833

[eva13197-bib-0060] Nørhave, N. J. , Spurgeon, D. , Svendsen, C. , & Cedergreen, N. (2012). How does growth temperature affect cadmium toxicity measured on different life history traits in the soil nematode *Caenorhabditis elegans*? Environmental Toxicology and Chemistry, 31(4), 787–793. 10.1002/etc.1746 22253140

[eva13197-bib-0061] Olson‐Manning, C. F. , Wagner, M. R. , & Mitchell‐Olds, T. (2012). Adaptive evolution: Evaluating empirical support for theoretical predictions. Nature Reviews Genetics, 13(12), 867–877. 10.1038/nrg3322 PMC3748133 23154809

[eva13197-bib-0062] Park, H. J. , Kim, W.‐Y. , Park, H. C. , Lee, S. Y. , Bohnert, H. J. , & Yun, D.‐J. (2011). SUMO and SUMOylation in plants. Molecules and Cells, 32(4), 305–316. 10.1007/s10059-011-0122-7 21912873 PMC3887640

[eva13197-bib-0063] Pelletier, F. , Clutton‐Brock, T. , Pemberton, J. , Tuljapurkar, S. , & Coulson, T. (2007). The evolutionary demography of ecological change: Linking trait variation and population growth. Science, 315(5818), 1571–1574. 10.1126/science.1139024 17363672

[eva13197-bib-0064] Qin, C.‐F. , He, M.‐H. , Chen, F.‐P. , Zhu, W. , Yang, L.‐N. , Wu, E.‐J. , Guo, Z.‐L. , Shang, L.‐P. , & Zhan, J. (2016). Comparative analyses of fungicide sensitivity and SSR marker variations indicate a low risk of developing azoxystrobin resistance in *Phytophthora infestans* . Scientific Reports, 6, 20483. 10.1038/srep20483. 26853908 PMC4745062

[eva13197-bib-0065] Rahmstorf, S. , & Coumou, D. (2011). Increase of extreme events in a warming world. Proceedings of the National Academy of Sciences, 108(44), 17905–17909. 10.1073/pnas.1101766108. PMC3207670 22025683

[eva13197-bib-0066] Rekanović, E. , Potočnik, I. , Milijašević‐Marčić, S. , Stepanović, M. , Todorović, B. , & Mihajlović, M. (2012). Toxicity of metalaxyl, azoxystrobin, dimethomorph, cymoxanil, zoxamide and mancozeb to *Phytophthora infestans* isolates from Serbia. Journal of Environmental Science and Health, 47(5), 403–409. 10.1080/03601234.2012.657043 22424065

[eva13197-bib-0067] Rodríguez‐Verdugo, A. , Gaut, B. S. , & Tenaillon, O. (2013). Evolution of *Escherichia coli* rifampicin resistance in an antibiotic‐free environment during thermal stress. BMC Evolutionary Biology, 13(1), 50. 10.1186/1471-2148-13-50 23433244 PMC3598500

[eva13197-bib-0068] Runno‐Paurson, E. , Hannukkala, A. O. , Kotkas, K. , Koppel, M. , Williams, I. H. , & Mänd, M. (2013). Impact of phytosanitary quality of seed potato and temporal epidemic progress on the phenotypic diversity of *Phytophthora infestans* populations. American Journal of Potato Research, 90(3), 245–254. 10.1007/s12230-013-9299-y

[eva13197-bib-0069] Sambandan, D. , Carbone, M. A. , Mackay, T. F. , & Anholt, R. J. G. (2008). Phenotypic plasticity and genotype by environment interaction for olfactory behavior in *Drosophila melanogaster* . Genetics, 179, 1079–1088. 10.1534/genetics.108.086769 18505870 PMC2429861

[eva13197-bib-0070] Saville, A. , Graham, K. , Grünwald, N. J. , Myers, K. , Fry, W. E. , & Ristaino, J. B. (2014). Fungicide Sensitivity of U.S. Genotypes of *Phytophthora infestans* to Six oomycete‐targeted compounds. Plant Disease, 99, 659–666. 10.1094/PDIS-05-14-0452-RE 30699679

[eva13197-bib-0071] Schade, F. M. , Shama, L. N. , & Wegner, K. M. (2014). Impact of thermal stress on evolutionary trajectories of pathogen resistance in three‐spined stickleback (*Gasterosteus aculeatus*). BMC Evolutionary Biology, 14(1), 164. 10.1186/s12862-014-0164-5 25927537 PMC4115170

[eva13197-bib-0072] Schepers, H. T. A. M. , Kessel, G. J. T. , Lucca, F. , Förch, M. G. , Van Den Bosch, G. B. M. , Topper, C. G. , & Evenhuis, A. (2018). Reduced efficacy of fluazinam against *Phytophthora infestans* in the Netherlands. European Journal of Plant Pathology, 151, 947–960. 30996524 10.1007/s10658-018-1430-y PMC6435203

[eva13197-bib-0073] Schwaegerle, K. E. , McIntyre, H. , & Swingley, C. (2000). Quantitative genetics and the persistence of environmental effects in clonally propagated organisms. Evolution, 54(2), 452–461. 10.1554/0014-3820(2000)054 10937222

[eva13197-bib-0074] Sharma, K. , Gossen, B. D. , & McDonald, M. R. (2011). Effect of temperature on cortical infection by *Plasmodiophora brassicae* and clubroot severity. Phytopathology, 101(12), 1424–1432. 10.1094/PHYTO-04-11-0124 21864086

[eva13197-bib-0075] Shuman, E. (2011). Global climate change and infectious diseases. New England Journal Medicine, 2(1), 1061–1063. 10.1056/NEJMp0912931 20335580

[eva13197-bib-0076] Spitze, K. (1993). Population structure in *Daphnia obtusa*: Quantitative genetic and allozymic variation. Genetics, 135(2), 367–374. 8244001 10.1093/genetics/135.2.367 PMC1205642

[eva13197-bib-0077] Stefansson, T. S. , McDonald, B. A. , & Willi, Y. (2014). The influence of genetic drift and selection on quantitative traits in a plant pathogenic fungus. PLoS One, 9(11), e112523. 10.1371/journal.pone.0112523 25383967 PMC4226542

[eva13197-bib-0078] Thind, T. S. (2012). Fungicide resistance in crop protection: Risk and management. Plant Pathology, 61, 820–820. 10.1111/j.1365-3059.2012.02623.x

[eva13197-bib-0079] Tian, Y. , Yin, J. , Sun, J. , Ma, H. , Ma, Y. , Quan, J. , & Shan, W. (2015). Population structure of the late blight pathogen *Phytophthora infestans* in a potato germplasm nursery in two consecutive years. Phytopathology, 105(6), 771–777. 10.1094/PHYTO-03-14-0073-R 25738550

[eva13197-bib-0080] Tonsor, S. J. , Elnaccash, T. W. , & Scheiner, S. M. (2013). Developmental instability is genetically correlated with phenotypic plasticity, constraining heritability, and fitness. Evolution, 67(10), 2923–2935. 10.1111/evo.12175 24094343

[eva13197-bib-0081] Tooley, P. W. , Browning, M. , Kyde, K. L. , & Berner, D. (2009). Effect of temperature and moisture period on infection of Rhododendron ‘Cunningham's White’by *Phytophthora ramorum* . Phytopathology, 99(9), 1045–1052. 10.1094/PHYTO-99-9-1045 19671006

[eva13197-bib-0082] Webber, M. , & Piddock, L. (2003). The importance of efflux pumps in bacterial antibiotic resistance. Journal of Antimicrobial Chemotherapy, 51(1), 9–11. 10.1093/jay/dkg050 12493781

[eva13197-bib-0083] Wu, E.‐J. , Yang, L.‐N. , Zhu, W. , Chen, X.‐M. , Shang, L.‐P. , & Zhan, J. (2016). Diverse mechanisms shape the evolution of virulence factors in the potato late blight pathogen *Phytophthora infestans* sampled from China. Scientific Reports, 6, 26182. 10.1038/srep26182 27193142 PMC4872137

[eva13197-bib-0084] Yang, L. , Gao, F. , Shang, L. , Zhan, J. , & McDonald, B. A. (2013). Association between virulence and triazole tolerance in the phytopathogenic fungus *Mycosphaerella graminicola* . PLoS One, 8(3), e59568. 10.1371/journal.pone.0059568 23555044 PMC3598747

[eva13197-bib-0085] Yang, L. , Ouyang, H. B. , Fang, Z. G. , Zhu, W. , Wu, E. J. , Luo, G. H. , Shang, L. P. , & Zhan, J. (2018). Evidence for intragenic recombination and selective sweep in an effector gene of *Phytophthora infestans* . Evolutionary Applications, 11(8), 1342–1353. 10.1111/eva.12629 30151044 PMC6099815

[eva13197-bib-0086] Yang, L. N. , Zhu, W. , Wu, E. J. , Yang, C. , Thrall, P. H. , Burdon, J. J. , Jin, L. P. , Shang, L. P. & Zhan, J. (2016). Trade‐offs and evolution of thermal adaptation in the Irish potato famine pathogen *Phytophthora infestans* . Molecular Ecology, 25(16), 4047–4058. 10.1111/mec.13727 27288627

[eva13197-bib-0087] Yeh, F. , Yang, R. , Boyle, T. , Ye, Z. , & Xiyan, J. (2000). POPGENE 32, microsoft windows‐based freeware for population genetic analysis. University of Alberta, Edmonton: Molecular biology and biotechnology centre.

[eva13197-bib-0088] Yu, M. , Song, W. , Tian, F. , Dai, Z. , Zhu, Q. , Ahmad, E. , Guo, S. , Zhu, C. , Zhong, H. , Yuan, Y. , Zhang, T. , Yi, X. , Shi, X. , Gan, Y. , & Gao, H. (2019). Temperature‐ and rigidity‐mediated rapid transport of lipid nanovesicles in hydrogels. Proceedings of the National Academy of Sciences, 116(12), 5362–5369. 10.1073/pnas.1818924116 PMC6431219 30837316

[eva13197-bib-0089] Zhan, J. , Linde, C. C. , Jurgens, T. , Merz, U. , Steinebrunner, F. , & McDonald, B. A. (2005). Variation for neutral markers is correlated with variation for quantitative traits in the plant pathogenic fungus *Mycosphaerella graminicola* . Molecular Ecology, 14(9), 2683–2693. 10.1111/j.1365-294X.2005.02638.x 16029470

[eva13197-bib-0090] Zhan, J. , & McDonald, B. A. (2011). Thermal adaptation in the fungal pathogen *Mycosphaerella graminicola* . Molecular Ecology, 20(8), 1689–1701. 10.1111/j.1365-294X.2011.05023.x 21395890

[eva13197-bib-0091] Zhan, J. , Stefanato, F. , & McDonald, B. A. (2006). Selection for increased cyproconazole tolerance in *Mycosphaerella graminicola* through local adaptation and in response to host resistance. Molecular Plant Pathology, 7(4), 259–268. 10.1111/j.1364-3703.2006.00336.x 20507445

[eva13197-bib-0092] Zhang, T. , Yang, Y. , & Pruden, A. (2015). Effect of temperature on removal of antibiotic resistance genes by anaerobic digestion of activated sludge revealed by metagenomic approach. Applied Microbiology and Biotechnology, 99(18), 7771–7779. 10.1007/s00253-015-6688-9 25994259

[eva13197-bib-0093] Zhu, W. , Shen, L.‐L. , Fang, Z.‐G. , Yang, L.‐N. , Zhang, J.‐F. , Sun, D.‐L. , & Zhan, J. (2016). Increased frequency of self‐fertile isolates in *Phytophthora infestans* may attribute to their higher fitness relative to the A1 isolates. Scientific Reports, 6, 29428. 10.1038/srep29428 27384813 PMC4935937

[eva13197-bib-0094] Zhu, W. , Yang, L.‐N. , Wu, E.‐J. , Qin, C.‐F. , Shang, L.‐P. , Wang, Z.‐H. , & Zhan, J. (2015). Limited sexual reproduction and quick turnover in the population genetic structure of *Phytophthora infestans* in Fujian, China. Scientific Reports, 5, 10094. 10.1038/srep10094 25970264 PMC4429539

