# Supplementary material for: Increasing temperature elevates the variation and spatial differentiation of pesticide tolerance in a plant pathogen
Source: Evol Appl. 2021 Feb 2;14(5):1274–85. doi: 10.1111/eva.13197 (PMC8127700; doi:10.1111/eva.13197)
Supplement: Supplementary file 1 — Table S1 [file EVA-14-1274-s001.docx]

**Supplementary Table 1**: Geographic coordinate and annual mean temperature (mean, variance and standard deviation) of the nine populations sampled for *Phytophthora infestans*.

| **Population** | **Location** | **Longitude** | **Latitude** | **Annual temperature** | | |
| --- | --- | --- | --- | --- | --- | --- |
|  |  |  |  | **Mean (°C )** | **Variance** | **SD*** |
| Inner Mongolia | Arong | 123°28' | 48°08' | -0.63 | 246.40 | 15.70 |
| Ningxia | Guyuan | 106°14' | 36°01' | 7.00 | 90.02 | 9.49 |
| Gansu | Tianshui | 105°43' | 34°35' | 11.70 | 79.33 | 8.91 |
| Guizhou | Anshhun | 105°56' | 26°16' | 14.70 | 41.20 | 6.42 |
| Yunnan | Kunming | 102°43' | 25°03' | 15.60 | 20.14 | 4.49 |
| Hubei | Wuhan | 114°13' | 30°02' | 17.00 | 78.61 | 8.87 |
| Xiapu | Xiapu | 119°59' | 26°54' | 20.30 | 45.97 | 6.78 |
| Fuzhou | Changle | 119°17' | 26°05' | 20.50 | 43.29 | 6.58 |
| Guangxi | Nanning | 108°22' | 22°50' | 22.60 | 34.17 | 5.85 |

*Standard deviation
